# Vaspin regulates the osteogenic differentiation of MC3T3-E1 through the PI3K-Akt/miR-34c loop

**DOI:** 10.1038/srep25578

**Published:** 2016-05-09

**Authors:** Yuan Liu, Feng Xu, Hong-Xia Pei, Xiao Zhu, Xiao Lin, Cheng-Yuan Song, Qiu-Hua Liang, Er-Yuan Liao, Ling-Qing Yuan

**Affiliations:** 1 Institute of Metabolism and Endocrinology, Second Xiang-Ya Hospital, Central South University, Changsha, Hunan, People’s Republic of China; 2 Department of Endocrinology, Qilu Hospital, Shandong University, Jinan, Shandong, People’s Republic of China; 3 Department of Neurology, Xiangtan Central Hospital, Xiangtan, Hunan, People’s Republic of China; 4 Department of Endocrinology, The Third Affilliated Hospital of Southern Medical University, Guangdong, People’s Republic of China

## Abstract

Vaspin (visceral adipose tissue-derived serine protease inhibitor) is a newly discovered adipokine that widely participates in diabetes mellitus, polycystic ovarian syndrome and other disorders of metabolism. However, the effect of vaspin on the regulation of osteogenesis and the mechanism responsible are still unclear. Here, we found that vaspin can attenuate the osteogenic differentiation of the preosteoblast cell line MC3T3-E1 in a dose-dependent way; also, during this process, the expression of miRNA-34c (miR-34c) was significantly increased. Down-regulation of the expression of miR-34c in MC3T3-E1 diminished the osteogenic inhibitory effect of vaspin, while the up-regulation of miR-34c increased this effect through its target gene Runx2. Meanwhile, we found that vaspin could also activate the PI3K-Akt signalling pathway. Blocking the PI3K-Akt signalling pathway with specific inhibitors could decrease the osteogenic inhibitory effect of vaspin as well as the expression level of miR-34c. Furthermore, knock-down of miR-34c could promote the activation of Akt, which was probably realised by targeting c-met expression. Thus, PI3K-Akt and miR-34c constituted a modulation loop and controlled the expression of each other. Taken together, our study showed that vaspin could inhibit the osteogenic differentiation *in vitro*, and the PI3K-Akt/miR-34c loop might be the underlying mechanism.

Fat is the largest endocrine organ in the human body and has a complex connection with bone. In the past, it was widely accepted that fat was a protective factor for bone mass. The loading effect caused by fat mass cooperates with that of muscle mass and can protect the bone mineral density (BMD) from declining^1^. However, recent studies have challenged the conventional standpoint which states that fat might be a beneficial factor for bone health. One study carried out in 197 elderly patients showed that fracture risk was positively related to the fat mass in the bone marrow^2^, and this viewpoint gained support from additional researches. According to these studies, negative associations between fat mass and bone tissue have been demonstrated in children, men, and pre-and post-menopausal women^3^,^4^,^5^,^6^,^7^,^8^,^9^.

Recently, visceral adipose tissue (VAT) has gained increasing attention on the basis of robust evidence linking VAT and insulin resistance, metabolic syndrome, dyslipidaemia, cardiovascular disease, hypertension, and cancer^10^,^11^,^12^,^13^,^14^. In addition, according to Cohen’s study, VAT has been shown to show the most significant association with lower BMD; post-menopausal women with more VAT showed a significant decline in both BMD and microarchitecture, and also the bone regeneration rate was greatly suppressed^15^. Furthermore, the similar result was found in Júnior’s study, who found that BMD was negatively correlated with VAT^16^. These results all indicated that VAT might have a negative effect on bone, and the adipokines might be responsible.

To date, many adipokines have been found to participate in bone metabolism. Most of them act as promoting factors on osteogenic differentiation, for instance, adiponectin^17^, visfatin^18^, omentin-1^19^,^20^ and resistin^21^. However, the effect of vaspin on osteogenic differentiation still needs to be illuminated.

Vaspin is a secretory protein which is mainly synthesised in the visceral fat tissue; however, its expression has also been reported in the liver, pancreas, and skin^22^,^23^. The expression of vaspin could be affected by many factors, including gender, age, obesity and so on^24^,^25^,^26^. Fazeli found that the serum vaspin concentration was significantly increased in patients with colorectal cancer^27^. Li and colleagues found that a single nucleotide polymorphism in the vaspin gene was closely related to coronary heart disease^28^. Kukla’s study showed that serum vaspin concentration could be a predictive factor of hepatic fibrosis^29^. This discovery was further confirmed by *in vitro* studies, where research found that vaspin could inhibit the osteoclastogenesis of RAW264.7 cells^30^; our previous study showed that vaspin could attenuate apoptosis in human osteoblasts through the ERK signalling pathway^31^. However, the effect of vaspin on osteogenesis is still unclear. Therefore, the aim of this study is to determine the effect and mechanisms of vaspin on the osteogenic differentiation of MC3T3-E1 cells, a well-defined pre-osteoblast in investigating osteogenesis^32^,^33^.

## Results

### Vaspin attenuated the osteogenic differentiation of MC3T3-E1

**Figure 1:**
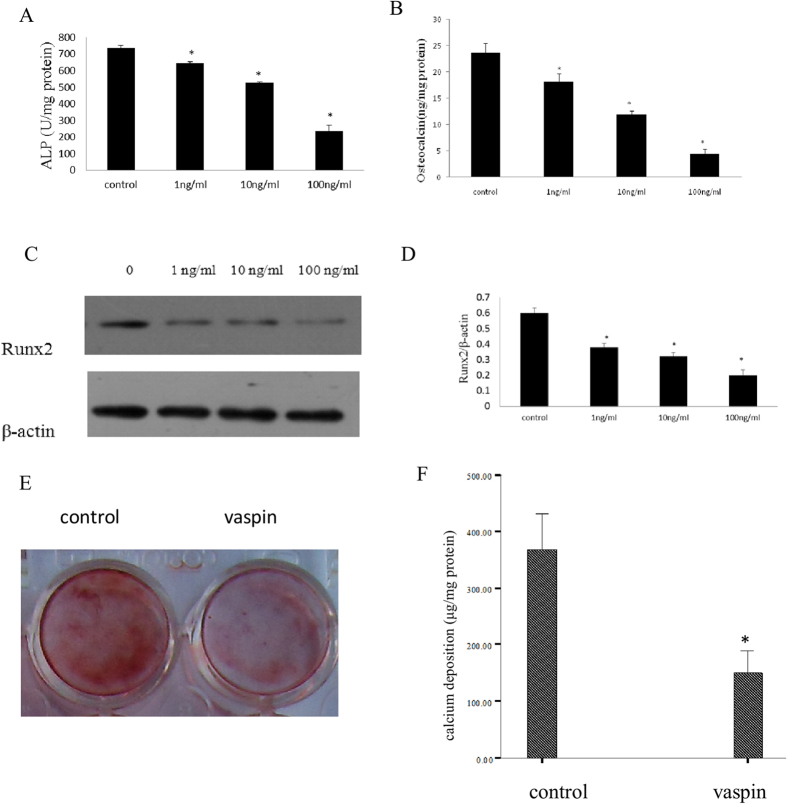
Effects of vaspin on the osteogenic differentiation of MC3T3-E1 cells. (**A**) Effect of vaspin on ALP activity. The cells were treated with vehicle or vaspin (1–100 ng/mL) for 48 h. ALP activity was measured by an ALP kit, normalised to the cellular protein contents. (**B**) Effect of vaspin on OC secretion. The cells were treated with vehicle or vaspin (1–100 ng/mL) for 48 h. OC secretion was determined by radioimmunoassay, normalised to the cellular protein contents. (**C,D**) Effect of vaspin on Runx2 protein expression. The cells were treated with vehicle or vaspin (1–100 ng/mL). The expression of Runx2 protein was measured by western blotting. The data are presented as densitometric ratios of Runx2/β-actin. (**E**) A representative entire plate view of the Alizarin Red S staining in 24-well plates for control cells and cells treated with100 ng/ml vaspin in 20-day cultures. (**F**)Quantification of Alizarin Red S stain via extraction with cetyl-pyridinium chloride. The amount of released dye was quantified by spectrophotometry at 540 nm. The bars represent the mean ± SD (n = 3; *p < 0.05 vs. control).

It is widely accepted that alkaline phosphatase (ALP) activity, osteocalcin (OC) secretion, and Runx2 protein expression are important markers of osteogenic differentiation. In the present study, we determined the alterations of these markers in premature osteoblasts (MC3T3-E1) after vaspin administration in order to determine the effect of vaspin on osteogenic differentiation. Our data demonstrated that treatment with vaspin significantly inhibited ALP activity and the suppression was dose-dependent. In MC3T3-E1 cells, significant inhibition by vaspin was first observed at 1 ng/ml and the inhibition effect peaked at 100 ng/ml. Details of the effects of vaspin on ALP activityin MC3T3-E1 cells are shown in Fig. 1A. OC secretion was determined by radioimmunoassay; Fig. 1B shows that vaspin significantly decreased the secretion of OC. The expression of Runx2 protein was determined by western blotting, which gave results similar to those of ALP activity and OC secretion in MC3T3-E1 cells. The expression of Runx2 was significantly inhibited after treatment with vaspin (Fig. 1C,D). Figure 1E shows an entire plate of cells stained with Alizarin Red S, indicating that the treatment of MC3T3-E1 cells with 100 ng/ml vaspin in culture for 20 days significantly decreased the level of mineralised nodule formation.

### MiR-34c expression was increased during vaspin administration

**Figure 2:**
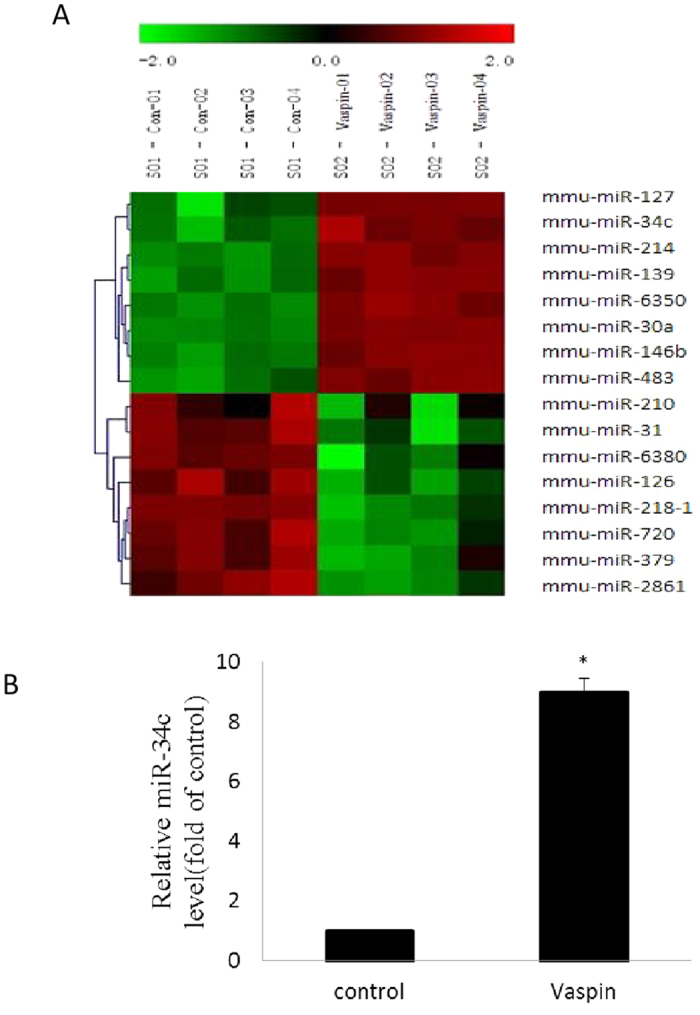
Microarray analysis of miRNA expression after vaspin administration in MC3T3-E1 cells. The cells were treated with or without 100 ng/ml vaspin. (**A**) Total RNA was isolated and expression of miRNAs was analysis by a microarray platform. (**B**) MiR-34c expression was determined by real-time quantitative polymerase chain reaction (qRT-PCR). Results are presented as fold of U6 expression as a control. Bars present mean ± SD (n = 3; *p < 0.05 vs. control).

To understand the miRNAs that are potentially involved in osteogenic differentiation after vaspin administration, we analysed the expression of miRNAs using an established microarray plat form that contained probe sequences for 1281 mature mouse miRNAs. Eight of them were up-regulated obviously and eight of them were down-regulated notably during the differentiation stage (Fig. 2A). From the most up-regulated miRNAs, miR-34c was selected for further investigation since previous studies have demonstrated that miR-34c might be involved in osteoblast differentiation^34^. The qRT-PCR results confirmed that the expression of miR-34c was elevated in MC3T3-E1 cells after vaspin administration (Fig. 2B).

### MiR-34c facilitates the inhibitory effect of vaspin on MC3T3-E1 osteogenic differentiation

**Figure 3:**
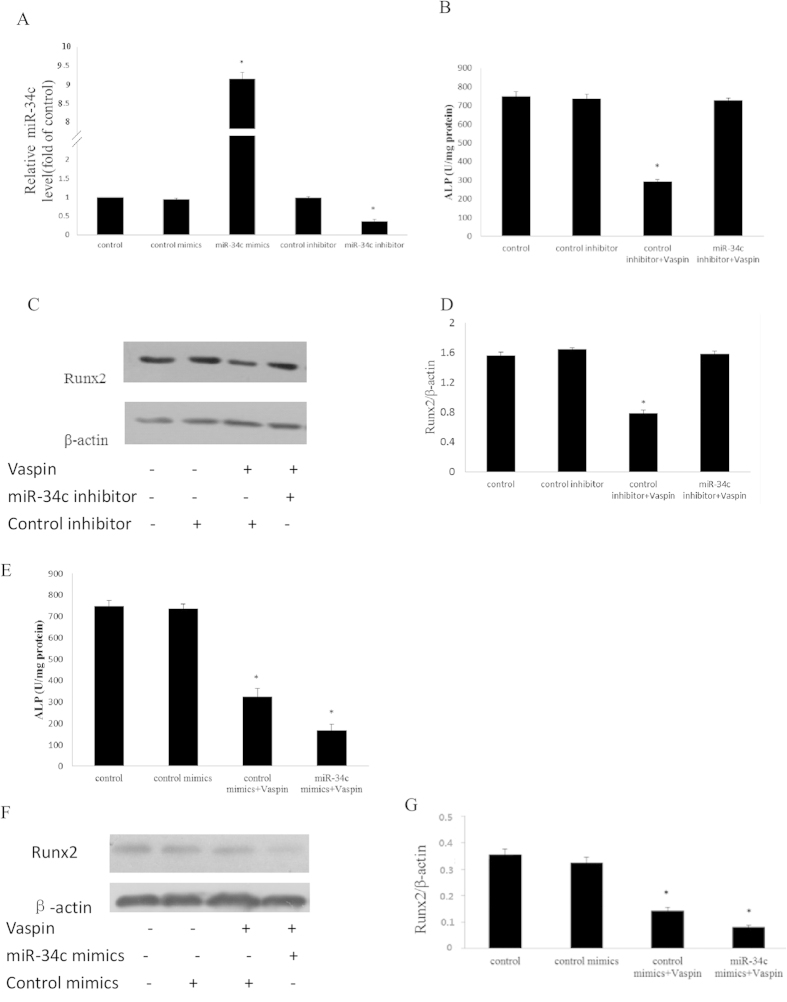
Effect of miR-34c during the regulation of vaspin on MC3T3-E1 cell osteogenic differentiation. (**A**) Over-expression and down-regulationof miR-34c in MC3T3-E1 cells. MC3T3-E1 cells were transfected with miR-34c mimics or miR-34c inhibitor or their control oligos, respectively. The expression of miR-34c was determined by qRT-PCR analysis. Results are presented as fold of U6 expression. (**B–D**) Effect of down-regulated miR-34c on the osteogenic differentiation of MC3T3-E1 cells treated with vaspin. MC3T3-E1 cells were transfected with control inhibitor or miR-34c inhibitor and then treated with 100 ng/ml vaspin for 48h, respectively. ALP activity was measured by an ALP kit, normalised to the cellular protein contents, and the Runx2 protein expression was measured by western blotting, respectively. The data are presented as densitometric ratios of Runx2/β-actin. (**E–G**) Effect of over-expressed miR-34c on the osteogenic differentiation of MC3T3-E1 cells treated with vaspin. MC3T3-E1 cells were transfected with control mimics or miR-34c mimics and then treated with 100 ng/ml vaspin, respectively. ALP activity was measured by an ALP kit, normalised to the cellular protein contents, and the Runx2 mRNA and protein expressions were measured by qRT-PCR and western blotting. The data are presented as densitometric ratios of Runx2/β-actin. The results are presented as mean ± SD (n = 3; *p < 0.05 vs. control).

To determine the role of miR-34c in vaspin-treated MC3T3-E1 cells, gain-of-function and loss-of-function models were employed. MC3T3-E1 cells were transfected with miR-34c mimics and inhibitors, and qRT-PCR confirmed the successful over-expression and down-regulation of miR-34c level (Fig. 3A). Then, the cells were cultured in the α-MEM medium with 100 ng/ml vaspin. Compared to the control group, the ALP activity and Runx2 expression were increased in the miR-34c down-regulation group (Fig. 3B–D), and decreased in the over-expressed group after vaspin administration (Fig. 3E–G). These results indicated that miR-34c facilitated the inhibitory effect of vaspin on MC3T3-E1 osteogenic differentiation.

### The target of miR-34c

**Figure 4:**
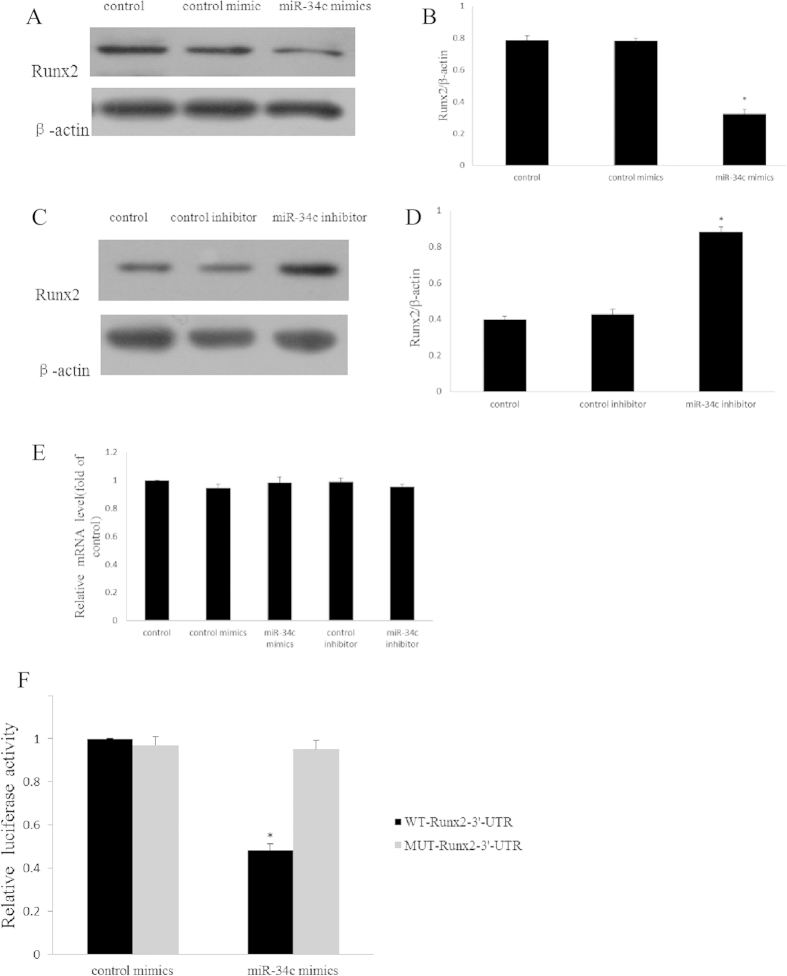
Runx2 is the target of miR-34c. (**A,B**) Influence of over-expressed miR-34c on Runx2 protein expression. MC3T3-E1 cells were transfected with scramble mimics or miR-34c mimics respectively, and the expression of Runx2 protein was determined by western blotting. The data are presented as densitometric ratios of Runx2/β-actin. (**C,D**) Influence of down-regulated miR-34c on Runx2 protein expression. MC3T3-E1 cells were transfected with control inhibitor or miR-34c inhibitor, respectively, and the expression of Runx2 protein was determined by western blotting. The data are presented as densitometric ratios of Runx2/β-actin. (**E**). Influence of miR-34c on Runx2 mRNA expression. MC3T3-E1 cells were transfected with control mimics or miR-34c mimics, control inhibitor or miR-34c inhibitor, respectively, and the expression of Runx2 mRNA was determined by qRT-PCR analysis. Results are presented as fold of control. (**F**) MC3T3-E1 cells were co-transfected with the luciferase reporter carrying WT-Runx2-3′-UTR or MUT-Runx2-3′-UTR, and miR-34c mimics or control mimics. The luciferase activity wasmeasured 48 h after transfection; the data are presented as ratios of Firefly luciferase values/Renilla luciferase values. The results are presented as mean ± SD (n = 3; *p < 0.05 vs. control).

As mentioned previously, vaspin administration could cause an increase of miR-34c as well as a decrease of Runx2 expression. Also, according to Zhang, there is a binding site for miR-34c in the 3′ UTR region of Runx2 mRNA^34^, so we suspect that Runx2 might be the target of miR-34c during the osteogenic differentiation modulated by vaspin. In order to confirm that, we used the miRNA mimics and inhibitors to build the over-expression and down-expression model, respectively. We found that the Runx2 protein expression was increased in the down-expression model while decreased in the over-expression model (Fig. 4A–D). However, the mRNA expression had no significant difference in all groups (Fig. 4E). Moreover, the luciferase reporter further certified that the Runx2 was the target of miR-34c. A luciferase reporter containing the wild-type (WT) or mutant (MUT) 3′-UTR coding sequences for Runx2 was generated and introduced with miR-34c mimics into MC3T3-E1. Overexpression of miR-34c significantly repressed the luciferase activity of the WT-3′-UTR of Runx2 reporter plasmids but not that of the MUT-Runx2-3′-UTR reporters. Control mimics didn’t affect the wild-type or mutant constructs, confirming the specificity of the action (Fig. 4F). These results confirmed that miR-34c is involved in the osteogenic regulation of vaspin by post-transcriptionally repressing the expression of Runx2.

### Vaspin activates the PI3K-Akt and ERK signalling pathways in MC3T3-E1 cells

**Figure 5:**
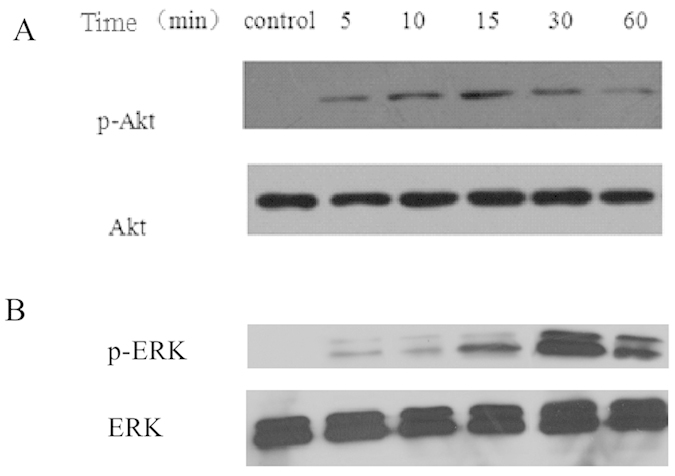
Effects of vaspin on PI3K-Akt and ERK activation in MC3T3-E1 cells. (**A**) Effect of vaspin on PI3K-Akt activation. MC3T3-E1 cells were exposed to 100 ng/ml vaspin for 0–60 min for Akt activation. Cell lysates were subjected to western blotting and incubated with antibodies against p-Akt, and Akt. (**B**) Effect of vaspin on ERK activation. MC3T3-E1 cells were exposed to 100 ng/ml vaspin for 0–60 min. Cell lysates were subjected to western blotting and incubated with antibodies against p-ERK, and ERK. The representative results are shown.

The PI3K-Akt and ERK signalling pathways play essential roles in the biological function of osteoblasts, such as the proliferation and apoptosis^35^,^36^. They are also involved in the osteogenic differentiation of MC3T3-E1 cells^37^,^38^,^39^. To clarify the effects of vaspin on activation of the PI3K-Akt and ERK signalling pathways in MC3T3-E1 cells, the expression of phosphorylated Akt (p-Akt) and phosphorylated ERK (p-ERK) was determined in the vaspin-treated MC3T3-E1 cells. Our data demonstrated that Akt was significantly phosphorylated by vaspin, and activation occurred 5 min after the start of incubation, peaking at 15 min (Fig. 5A). Similar results were observed in the ERK signalling pathway, which showed that ERK was phosphorylated by vaspin, and the activation occurred 5 min after vaspin treatment, peaking at 30 min (Fig. 5B).

### The PI3K-Akt signalling pathway inhibitor LY294002 attenuates the osteogenic inhibitory effect of vaspin

**Figure 6:**
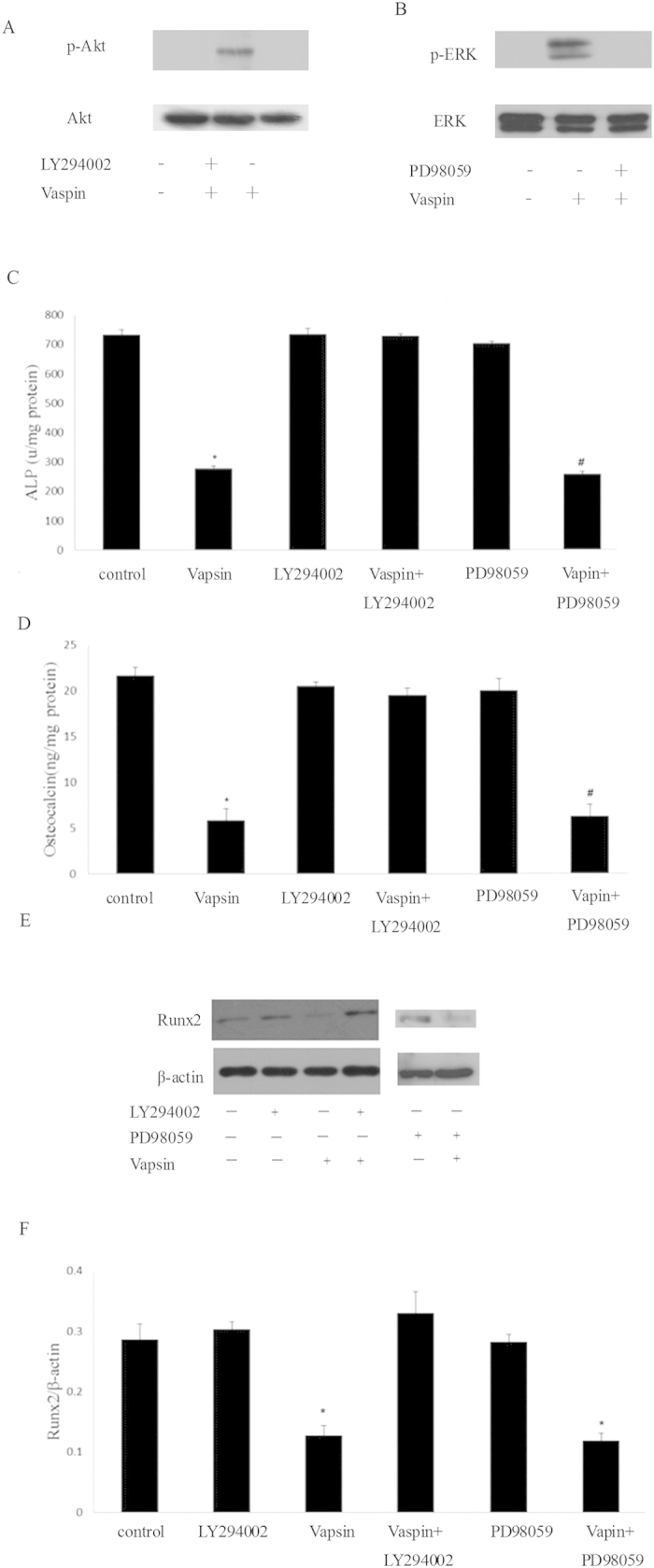
The PI3K-Akt signalling pathway mediates the inhibitory effect of vaspin on osteogenic differentiation of MC3T3-E1 cells. (**A**) Blocking of the PI3K-Akt signalling pathway with LY294002. MC3T3-E1 cells were incubated with LY294002 for 2 h prior to treatment with 100 ng/ml of vaspin for 15 min. Total proteins were subjected to western blotting and incubated with antibody against p-Akt and Akt. (**B**) Blocking of the ERK signalling pathway with PD98059. MC3T3-E1 cells were incubated with PD98059 for 2 h prior to treatment with 100 ng/ml of vaspin for 30 min. Total proteins were subjected to western blotting and incubated with antibody against p-ERK and ERK. The representative results are shown. (**C–F**) The PI3K-Akt pathway mediated the inhibitory effect of vaspin during the osteogenic differentiation of MC3T3-E1 cells, but the EKR signalling pathway did not. MC3T3-E1 cells were incubated with LY294002 or PD98059 for 2 h prior to treatment with 100 ng/ml of vaspin. The ALP activation (**C**), OC secretion (**D**) and Runx2 protein expression (**E,F**) were measured. The bar indicates mean ± SD (n = 3; *p < 0.05 vs. control).

To further confirm the effect of the PI3K-Akt and ERK signalling pathway during the osteogenic differentiation downstream regulation of vaspin, we used the specific inhibitor of the PI3K-Akt signalling pathway, LY294002, and the ERK signalling pathway, PD98059, to pre-treat the MC3T3-E1 cells before treatment with vaspin. Figure 6A,B showed that pre-treatment with LY294002 or PD98059 could diminish the activation of Akt or ERK caused by vaspin, respectively. Moreover, after incubating with vaspin, MC3T3-E1 cells pre-treated with LY294002 showed a higher level of ALP activity, OC secretion and Runx2 protein expression (Fig. 6B–E). However, pre-treatment with PD98059 did not attenuate the effect of vaspin on ALP activity, OC secretion and Runx2 expression (Fig. 6B–E). This result indicates that the PI3K-Akt signalling pathway participates in the osteogenic modulation of vaspin, but the ERK signalling pathway does not.

### The PI3K-Akt signalling pathway inhibitor LY294002 attenuates the expression of miR-34c

**Figure 7:**
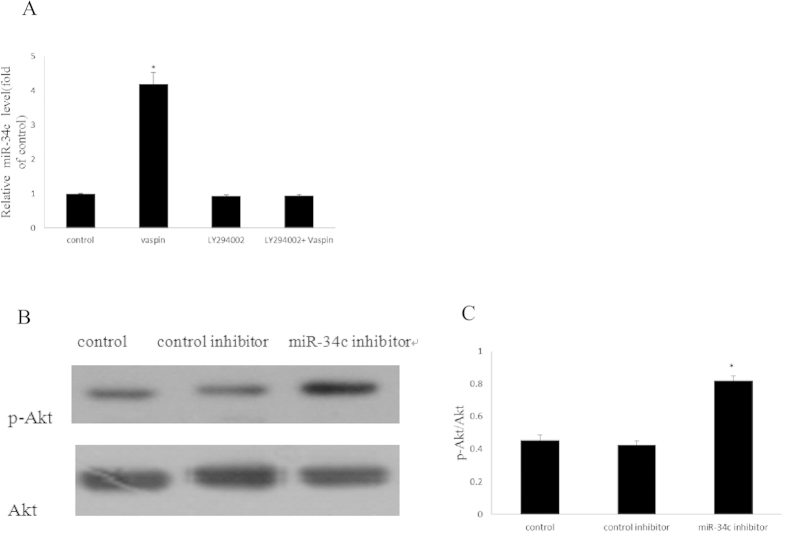
The relationship between Akt and miR-34c. (**A**) Effect of PI3K-Akt signalling pathway on the expression of miR-34c. MC3T3-E1 cells were incubated with or without LY294002 for 2 h prior to treatment with 100 ng/ml of vaspin. miR-34c expression was determined by qRT-PCR. Results are presented as fold of U6 expression. (**B,C**) Effect of miR-34c on activation of the PI3K-Akt signalling pathway. MC3T3-E1 cells were transfected with control inhibitor or miR-34c inhibitor respectively, and expression of the p-Akt protein was determined by western blotting. The data are presented as densitometric ratios of p-Akt/Akt. The bar indicates mean ± SD (n = 3; *p < 0.05 vs. control).

Evidence proved that numerous signalling pathways are involved in the modulation of miRNA expression, such as the TGF-β, PI3K-Akt, and ERK pathways^40^. In our present study, we found that vaspin could activate the PI3K-Akt signalling pathway and increase the expression of miR-34c at the same time. This result suggested that there was a correlation between the PI3K-Akt pathway and miR-34c expression. To further investigate the effect of the PI3K-Akt signalling pathway on the expression of miR-34c downstream of vaspin, we used LY294002 to pre-treat MC3T3-E1 cells. After incubating with vaspin, MC3T3-E1 cells pre-treated with LY294002 showed a decrease in the expression of miR-34c compared to the group without pre-treatment (Fig. 7A). Therefore, miR-34c might be a downstream element of the PI3K-Akt signalling pathway in this procedure, and blocking the PI3K-Akt pathway could decrease the expression of miR-34c and diminish the inhibitory effect of vaspin on the osteogenic differentiation of MC3T3-E1 cells as a result.

### MiR-34c alleviates the activation of the PI3K-Akt signalling pathway

To clarify the effect of miR-34c on activation of the PI3K-Akt signalling pathway, we investigated the level of p-Akt in the miR-34c down-expression model. We found that after transfection of the miR-34c inhibitor, miR-34c expression was remarkably decreased when compared to the control group (Fig. 3A). Meanwhile, the p-Akt level was significantly increased after transfection of the miR-34c inhibitor (Fig. 7B,C). This result indicates that miR-34c can inhibit activation of the PI3K-Akt signalling pathway.

### MiR-34c attenuates the expression of c-met

c-met is a transmembrane tyrosine kinase receptor which can phosphorylate the substrate and activate multiple signalling pathways such as PI3K-Akt and MAPK^41^,^42^. Meanwhile, c-met is also the target gene of miR-34c^43^,^44^,^45^; the over-expression of miR-34c could inhibit the c-met level and vice versa. Therefore, we assumed that c-met might be the pivot that combines the PI3K-Akt pathway and miR-34c in MC3T3-E1 cells.

**Figure 8:**
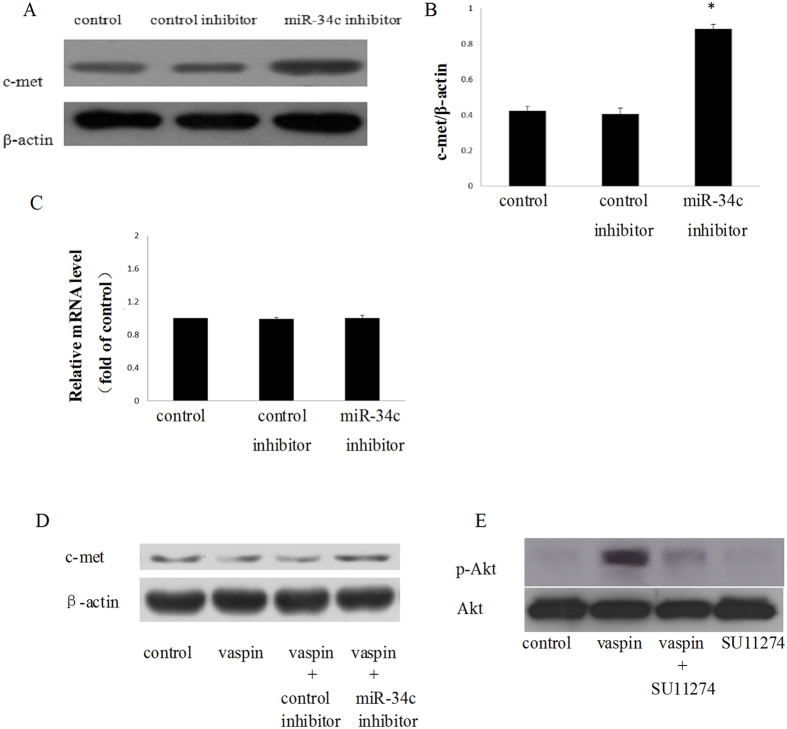
C-met mediates the vaspin-induced phosphorylation of Akt. (**A,B**) Effect of miR-34c on c-met protein expression. MC3T3-E1 cells were transfected with control inhibitor or miR-34c inhibitor respectively, and the expression of c-met protein was determined by western blotting. The data are presented as densitometric ratios of c-met/β-actin. (**C**) Effect of miR-34c on c-met mRNA expression. MC3T3-E1 cells were transfected with control inhibitor or miR-34c inhibitor, respectively, and the expression of c-met mRNA was determined by qRT-PCR analysis. Results are presented as fold of control. (**D**) The MC3T3-E1 cells were treated with vaspin, and the cells transfected with miR-34c inhibitor or its control were treated with 100 ng/ml vaspin; the expression of c-met protein was determined by western blotting. (**E**) The MC3T3-E1 cells were incubated with SU11274 for 2 h prior to treatment with 100 ng/ml of vaspin; cell lysates were subjected to western blotting and incubated with antibodies against p-Akt, and Akt. The representative results are shown. The results arepresented as mean ± SD (n = 3; *p< 0.05 vs. control).

To confirm this hypothesis, we determined the expression of c-met in the miR-34c down-expression model. We found that down-expression of miR-34c could increase expression of the c-met protein instead of c-met mRNA (Fig. 8A–C). Meanwhile, the expression of c-met dramatically decreased after treatment with vaspin in MC3T3-E1 cells, and this effect could be attenuated by knockdown of miR-34c (Fig. 8D). Thus, the variation trend of c-met protein was consistent with the p-Akt level.

To further confirm the effect of c-met on the activation of Akt, we used SU11274, an inhibitor of c-met, to observe whether c-met mediates the effect of vaspin on Akt. After blocking c-met, we found that p-Akt was significantly decreased (Fig. 8E). This result indicates that c-met might be the central junction of miR-34c and p-Akt.

## Discussion

In the present study, we found that vaspin could attenuate the osteogenic differentiation of preosteoblasts MC3T3-E1 cells in a dose-dependent manner, and the PI3K-Akt/miR-34c regulation loop might be the mechanism involved.

So far, numerous adipokines have been found, such as adiponectin, leptin, resistin, omentin, vaspin etc. They participate in multiple biological processes and play pivotal roles in human homeostasis. However, the relationship between those adipokines and bone metabolism is still unclear^46^. For example, adiponectin was found to have a promoting effect on osteogenic differentiation, but in the clinical investigations, the serum adiponectin concentration was negatively related to BMD^47^,^48^. Moreover, some studies have found that leptin could promote osteogenesis, while others showed its negative effect on bone mass; this paradoxical result is partly due to the different methods of administration^49^,^50^. Also, the role of some adipokines, such as vaspin and chemerin, in bone metabolism is still unclear. Here, our study showed for the first time that vaspin could attenuate the osteogenic differentiation of MC3T3-E1 in a dose-dependent manner; it is very important for us to have a better understanding of the relationship between adipokines and bone metabolism.

MiR-34c is a member of the miR-34 family and has been widely studied in oncogenesis, kidney fibrosis, Alzheimer’s disease and so on^51^,^52^,^53^. In past studies, lots of evidence has confirmed the critical role of miR-34c on bone metabolism. Palmieri found that the expression of miR-34c elevated in the Anatase coating induced bone formation^54^. Bae and colleagues also found that miR-34c was significantly increased in BMP-2-induced osteoblastic differentiation^55^. *In vivo*, osteoblast-specific gain of miR-34c in mice led to a decrease of osteoblastogenesis and caused age-dependent osteoporosis as the final result^55^. Our results demonstrated that miR-34c was expressed in MC3T3-E1 cells and its expression was greatly increased after vaspin administration. This finding suggested that miR-34c might be responsible for vaspin modulating the osteogenic differentiation of MC3T3-E1 cells. This suggestion was further confirmed by loss-of-function and gain-of-function experiments. Repression of the miR-34c level could attenuate the inhibitory effect of vaspin on osteogenesis and vice versa. These results all indicated that vaspin might inhibit osteogenic differentiation by elevating miR-34c expression.

MicroRNAs regulate biological activities through modulation of target genes. The seed region at the 5′ end of a miRNA can bind to the 3′UTR site of its target mRNA through sequence-specific base pairing and attenuate gene expression as a result. According to the Targetscan website, 516 genes are forecasted to be the putative targets of miR-34c, many of which have been identified by experiments. Runx2 is a transcription factor that plays a critical role in osteogenesis. Runx2 modulates the expression of multiple bone-related genes such as type I collagen, OC, and bone sialoprotein^56^. Also, Komori and colleagues found a complete lack of bone formation in Runx2-deficient mice due to the arrest of osteoblast maturation^57^. These findings confirmed the indispensible status of Runx2 in osteogenesis.

The previous studies have identified Runx2 as a target of miR-34c in MC3T3-E1 and osteosarcoma cells^34^,^58^. In our experiment, we found that the Runx2 level was decreased while miR-34c increased after vaspin administration. These all suggested that Runx2 might be the target of miR-34c in the osteogenic regulation of vaspin. Therefore, we briefly evaluated the effect of miR-34c on Runx2 expression. Our results showed that over-expression of miR-34c could attenuate the expression of the Runx2 protein, while low expression of miR-34c could promote the expression of Runx2. However, the mRNA level of Runx2 was not different between the groups; the luciferase reporter assay further confirmed this hypothesis. Taken together, these results suggest that miR-34c regulates the osteogenic differentiation downstream of vaspin via suppressing Runx2 at the post-transcriptional level. However, the mechanism by which vaspin elevates the level of miR-34c is still unknown and further investigation is needed.

There are two main procedures in the biogenesis of miRNAs: the cleaving of pri-miRNA into pre-miRNA in the nucleus and the cleaving of pre-miRNA hairpin into mature miRNA in cytoplasm. During the procedure, the RNase III enzymes Drosha and Dicer play essential roles^59^. The transcription and cleaving process of mature miRNA is a complex and delicate network that is strictly governed by multiple sets of molecules and signalling pathways such as the TGF-β, PI3K-Akt, and ERK^40^. To prove whether a signalling pathway is directly associated with miR-34c expression, we investigated the widely studied osteogenic-related signalling pathways PI3K-Akt and ERK after vaspin administration. The western blotting results demonstrated that incubating with vaspin led to activation of the PI3K-Akt and ERK signalling pathways in MC3T3-E1 cells. However, our previous study demonstrate vaspin could activate the ERK signaling pathway, but not Akt signaling pathway in human osteoblast^31^. We think the difference between different cell might be a result of different cells having different physical characteristics. MC3T3-E1 is immortalized osteoblast precursor cell line, which is a good model for studying osteoblast differentiation *in vitro*. However, human osteoblast is mature osteoblast, which is a good model for studying osteoblast physiology. Our result also showed blocking the activation of PI3K-Akt with LY294002 abolished the effect of vaspin on the osteogenic differentiation of MC3T3-E1 cells, but this was not true with the inhibitor of ERK. According to the research by Briata, the PI3K-Akt pathway intervenes in biogenesis by phosphorylating the KH-type splicing regulatory protein (KSRP). The phosphorylated KSRP binds with high affinity to the Drosha complex and facilitates the maturation of miRNA precursors^60^. In the present study, we found that activation of the PI3K-Akt signalling pathway was consistent with the increasing expression of miR-34c, while blocking the PI3K-Akt pathway with its specific inhibitor LY294002 could reduce the expression of miR-34c, suggesting that vaspin elevated the miR-34c level through activation of the PI3K-Akt signalling pathway.

Interestingly, we also found that the low expression of miR-34c can induce activation of the Akt pathway by increasing the p-Akt level. However, as we know, the miRNA participates in the biological procedures mainly by post-transcriptionally modulating the gene expression instead of via phosphorylation. Therefore, the strongest explanation was that there might be another target of miR-34c involved in this procedure which had tyrosine kinase activity. According to previous articles, c-met was a possible candidate.

C-met is the key factor in the HGF/c-met pathway, and is a transmembrane tyrosine kinase receptor that consists of two subunits: the α-subunit and β-subunit. The end of the β-subunit acts as a catalytic domain, mediating phosphorylation of the substrate and leading to downstream activation of the MAPK, PI3K-Akt and STAT3 signalling pathways and so on^42^,^61^. Previous studies have already shown that c-met signalling was regulated by several miRNAs including miR-34c^44^,^62^. Therefore, we suspect that c-met might be the hub of the miR-34c and PI3K-Akt signalling pathway. In order to confirm our hypothesis, we used a loss-of-function model. Our results showed that low expression of miR-34c could promote c-met protein expression which is consistent with the p-Akt level. Furthermore, our experiment confirmed that blocking c-met significantly attenuated the stimulatory effect of vaspin on the phosphorylation of Akt. Therefore, miR-34c might modulate the level of p-Akt by regulating the expression of c-met.

**Figure 9:**
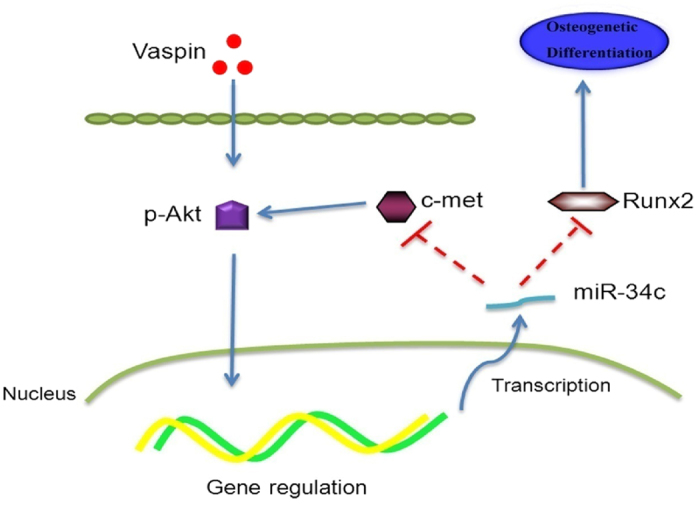
Vaspin modulates the osteogenic differentiation of MC3T3-E1 cells through PI3K-Akt/miR-34c loop.

The effect model of miRNA is not simply “one to one”; actually, one protein may be modulated by several miRNAs while one miRNA can regulate the expression of several proteins. In our study, we found that miR-34c could regulate osteogenic differentiation by targeting two targets: Runx2 and c-met. On the one hand, vaspin-induced miR-34c elevation could decrease expression of the Runx2 protein, leading to a decline in osteogenic differentiation as a result. On the other hand, increased miR-34c expression could target the c-met protein to reduce the activation of Akt; this feedback regulation was a protective method that promised the inhibitory effect within a controllable range. Taken together, vaspin elevated miR-34c expression through activation of the PI3K-Akt pathway, while miR-34c modulated activation of the PI3K-Akt signalling pathway by targeting c-met. These results confirmed a regulatory loop of the miR-34c/PI3K-Akt pathway that participates in the osteogenic modulation downstream of vaspin (Fig. 9).

In conclusion, the present study provided evidence for the first time that vaspin could inhibit the osteogenic differentiation of MC3T3-E1 cells and the novel PI3K-Akt/miR-34c regulatory loop was the possible mechanism involved. Our findings provide a better understanding of the relationship between obesity and osteoporosis.

## Materials and Methods

### Reagents

Recombinant mouse vaspin was purchased from the Enzo Life Sciences Inc. (Farmingdale, NY, USA). Antibodies for Runx2, β-actin, Akt, p-Akt, and c-met were purchased from Santa Cruz Biotechnology Inc. (Waltham, MA, USA). Akt inhibitor LY294002 was purchased from Calbiochem Corp. (San Diego, CA, USA). MiR-34c mimics and miR-34c inhibitors and their control oligos were purchased from Ribobio Co., Ltd (Guangzhou, China). The growth medium and foetal bovine serum (FBS), penicillin, and streptomycin were purchased from Gibco-BRL Co., Ltd (Grand Island, NY, USA). Ascorbic acid and β-glycerophosphate (β-GP), and Alizarin red S were purchased from Sigma Chemical Co., Ltd (St.Louis, MO, USA). All the experimental procedures were approved by the Ethics Committee of the Second Xiangya Hospital of Central South University, China and carried out in accordance with the approved guidelines.

### Cell cultures

The mouse preosteoblast cell line MC3T3-E1 (ATCC, Manassas, VA, USA) was maintained in α-minimum essential medium (α-MEM) supplemented with 10% FBS, 100 units/mL penicillin, and 100 mg/mL streptomycin in a humidified 5% CO_2_ atmosphere at 37 °C. The culture medium was changed every 2 days. Vaspin-free FBS was taken from the passage of FBS through Sepharose 4B affinity columns with the anti-vaspin antibody. Vaspin-free FBS was confirmed by western blot. To investigate the dose-dependent effect of vaspin on osteogenic differentiation of MC3T3-E1, 1 ng/ml to 100 ng/ml of vaspin was used to incubate the cells in the following experiments. The concentration of vaspin used was based on previous studies^31^. To explore the downstream cell signalling pathways involved in vaspin treatment, MC3T3-E1 cells were pre-treated with 10 μM PD98059 (an ERK inhibitor) or 10 μM LY294002 (a PI3K inhibitor) for 2 h prior to vaspin treatment.

### Analysis of alkaline phosphatase activity, osteocalcin secretion

ALP activity, osteocalcin secretion were measured as previously described^63^,^64^. Briefly, the cell layers were scraped into a solution containing 20 mM Tris–HCl (pH8.0), and 150 mM NaCl, 1% TritonX-100, 0.02% NaN3 and 1 mM PMSF. After the lysates were homogenised by sonication for 20 s, the alkaline phosphatase (ALP) activity was measured following the instructions provided with the ALP kit. Osteocalcin (OC) secretion in the culture media was measured with a specific radioimmunoassay kit (DiaSorin Corp., Stillwater, MN, USA) according to the manufacturer’s instructions. Protein expression was normalised to total cellular protein by Bradford protein assay.

### Measurement of mineralised matrix formation

For the induction of mineralisation, MC3T3-E1 cells were cultured in mineralisation-inducing medium, α-MEM supplemented with 50 mg/L ascorbic acid and 10 mM β-GP, with either 100 ng/ml vaspin or vehicle for 20 days. Then, the extent of mineralised matrix was determined by Alizarin Red S staining. Briefly, cells were fixed in 70% ethanol for 1 h at room temperature and stained with 40 mM Alizarin Red S for 10 min. Next, cell preparations were washed three times with PBS to eliminate nonspecific staining.

For the quantification of calcium levels, cells were rinsed in PBS and decalcified with 0.6 N HCl for 24 h. Calcium content was determined through a reaction with o-cresolphthalein, and calcium content of the cell layer was normalised to the total protein content using the Bradford protein assay.

### Microarray analysis of miRNAs

MC3T3-E1 cells were cultured in the absence or presence of 100 ng/ml vaspin. Then, the mirVana miRNA Isolation Kit (Ambion) was used for miRNA extraction. The μParaflo^TM^ microRNA microarray V19.0 which contained probe sequences based on the Sanger miRBase database release 19.0 (http://www.mirbase.org) was used for the examination of miRNA expression. Hybridisation was detected by fluorescence labelling with tag-specific Cy3 and Cy5 dyes. Microarray procedures and data analysis were performed as described^65^. For miRNA quantitation, the fold-change of the treatment group was obtained by normalising log 2 fluorescence with log 2 fluorescence of the control group. The normalised fold changes were analysed by dChip software (http://dchip-surv.chenglilab.org/;Boston, MA, USA). The results were confirmed by qRT-PCR.

### qRT-PCR analysis

Total RNA was extracted by Trizol (Invitrogen, CA, USA), and then cDNA was prepared using a cDNA Synthesis Kit (Thermo Scientific, PA, USA). The PCR was performed with Maxima SYBR Green qPCR Master Mix (Thermo Scientific, PA, USA). All procedures were strictly performed following the instructions provided. Amplification and detection were performed as follows: 50 °C for 2 min, 95 °C for 10 min and then 40 cycles of 95 °C for 15 s, 60 °C for 30 s, and 72 °C for 30 s. The results of qRT-PCR were automatically analysed by the Roche Light-Cycler technology. β-actin was used as the internal control of mRNA, and U6 was the control for miRNA in this experiment.

The miR-34c primer (miRQ0004580-1-2) and U6 primer (MQP-0201) used in this study were purchased from Ribobio Co., Ltd (Guangzhou, China)

### Detection of Runx2 and c-met in MC3T3-E1 cells by immunoblot analysis

To investigate the expression of Runx2 and c-met in MC3T3-E1 cells treated with vaspin, the cells layers were homogenised with RIPA lysate (Beyotime, Shanghai, China) and normalisedby BCA kit (Beyotime, Shanghai, China). Equal amounts of protein were submitted to SDS-PAGE and transferred onto 0.45 mm PVDF membranes (Pall, USA) to be stained with appropriate antibodies: anti-Runx2 (dilution1:200), -c-met (dilution 1:200) and -β-actin (dilution 1:400) antibodies. The reaction was visualised by chemiluminescence using an ECL kit (Thermoscientific Pierce, USA).

### Detection of ERK1/2 and Akt activation in MC3T3-E1 cell

To determine the effects of vaspin on activation of the signalling pathway, western blotting analysis was performed as above using anti-p-ERK, -ERK, -p-Akt, and -Akt antibodies, as described above.

### Cell transfection

[Table t1]For transient transfection of miR-34c mimics or miR-34c inhibitors, a combination of oligos and Lipofectamine 2000 were mixed gently following the manufacturer’s instructions and added to cells in 6-well plates at a density of 1 × 10^6^ cells per well. The medium was changed back to growth medium with or without 100 ng/ml vaspin 6 h after transfection.

### Luciferase reporter assay

To construct wild-type 3′-UTR (WT-pGL3), a segment of mouse Runx2 gene was amplified from mouse genomic DNA and inserted into the XbaI-FseI site in the pGL3-Control Firefly Luciferase reporter vector. To construct mutant 3′-UTR (MUT-pGL3), the QuickChange site-directed mutagenesis kit (Stratagene) was used to induce the point mutation in the UTR region of WT-pGL3. Plasmid DNA was sequenced for authenticity. The PCR and mutagenic primer sequences are shown in Table 2.

MC3T3-E1 cells were co-transfected with luciferase reporter carrying WT-pGL3 or MUT-pGL3 and miR-34c mimics or control mimics. Then, 48 hours after transfection, luciferase activity in each group was detected with the luciferase assay system (Promega).

### Statistical analysis

Results were presented as mean ± standard deviation (SD), and analysis was performed with Statistical Product and Service Solutions (v18.0). Differences between groups were evaluated by one-way analysis of variance (ANOVA). The data shown were based on three independent experiments. A level of p < 0.05 was considered significant.

## Additional Information

**How to cite this article**: Liu, Y. *et al*. Vaspin regulates the osteogenic differentiation of MC3T3-E1 through the PI3K-Akt/miR-34c loop. *Sci. Rep.* **6**, 25578; doi: 10.1038/srep25578 (2016).

## Figures and Tables

**Table 1 t1:** The primers for mRNA used in this study were as follows.

Gene name	pimer	
Runx2	5′-CACTGGCGGTGCAACAAGA-3′	
	5′-TTTCATAACAGCGGAGGCATTTC-3′	
c-met	5′-CACTGGCGGTGCAACAAGA-3′	
	5′-TTTCATAACAGCGGAGGCATTTC-3′	
β-actin	5′-GCGTGACATCAAAGAGAAGC-3′	
	5′-AGCACTGTGTTGGCATAGAG-3′	

**Table 2:** Nucleotide sequences of primers for construct and mutation of plasmids.

Name	Primers
WT Runx2	5′-GCTCTAGAGGCCAGCCAAGGATGCCAGAC-3′
	5′-GGCCGGCCAACCAACCCAGGGCGGAGA-3′
MUT Runx2	5′-GCGGAGAGACCATGAAGAAAATACTTTAG
	TGGTGATAAAAATAAATTTTGGATTTCAATTTCTTGA-3′
	5′-TCAAGAAATTGAAATCCAAAATTTATTTTTATCA
	CCACTAAAGTATTTTCTTCATGGTCTCTCCGC-3′

## References

[b1] Hinriksdottir G., Arngrimsson S. A., Misic M. M. & Evans E. M. Lean soft tissue contributes more to bone health than fat mass independent of physical activity in women across the lifespan. Maturitas 74, 264–269, doi: 10.1016/j.maturitas.2012.12.009 (2013). 23313436

[b2] Schwartz A. V. . Vertebral bone marrow fat associated with lower trabecular BMD and prevalent vertebral fracture in older adults. J Clin Endocrinol Metab 98, 2294–2300, doi: 10.1210/jc.2012-3949 (2013). 23553860 PMC3667265

[b3] Leslie W. D. . Estimated lean mass and fat mass differentially affect femoral bone density and strength index but are not FRAX independent risk factors for fracture. J Bone Miner Res 29, 2511–2519, doi: 10.1002/jbmr.2280 (2014). 24825359

[b4] Bredella M. A. . Vertebral bone marrow fat is positively associated with visceral fat and inversely associated with IGF-1 in obese women. Obesity (Silver Spring) 19, 49–53, doi: 10.1038/oby.2010.106 (2011). 20467419 PMC3593350

[b5] Gilsanz V. . Reciprocal relations of subcutaneous and visceral fat to bone structure and strength. J Clin Endocrinol Metab 94, 3387–3393, doi: 10.1210/jc.2008-2422 (2009). 19531595 PMC2741723

[b6] Pollock N. K. . Bone and fat relationships in postadolescent black females: a pQCT study. Osteoporos Int 22, 655–665, doi: 10.1007/s00198-010-1266-6 (2011). 20449571

[b7] Janicka A. . Fat mass is not beneficial to bone in adolescents and young adults. J Clin Endocrinol Metab 92, 143–147, doi: 10.1210/jc.2006-0794 (2007). 17047019

[b8] Nielson C. M. . BMI and fracture risk in older men: the osteoporotic fractures in men study (MrOS). J Bone Miner Res 26, 496–502, doi: 10.1002/jbmr.235 (2011). 20814955 PMC3179296

[b9] Hsu Y. H. . Relation of body composition, fat mass, and serum lipids to osteoporotic fractures and bone mineral density in Chinese men and women. Am J Clin Nutr 83, 146–154 (2006). 16400063 10.1093/ajcn/83.1.146

[b10] Smith U. Abdominal obesity: a marker of ectopic fat accumulation. J Clin Invest 125, 1790–1792, doi: 10.1172/JCI81507 (2015). 25932676 PMC4463217

[b11] Fischer K., Pick J. A., Moewes D. & Nothlings U. Qualitative aspects of diet affecting visceral and subcutaneous abdominal adipose tissue: a systematic review of observational and controlled intervention studies. Nutr Rev 73, 191–215, doi: 10.1093/nutrit/nuu006 (2015). 26024544

[b12] Breitfeld J. . Role of vaspin in human eating behaviour. PLos One 8, e54140, doi: 10.1371/journal.pone.0054140 (2013). 23342091 PMC3544656

[b13] Phalitakul S., Okada M., Hara Y. & Yamawaki H. A novel adipocytokine, vaspin inhibits platelet-derived growth factor-BB-induced migration of vascular smooth muscle cells. Biochem Biophys Res Commun 423, 844–849, doi: 10.1016/j.bbrc.2012.06.052 (2012). 22713468

[b14] Tchernof A. & Despres J. P. Pathophysiology of human visceral obesity: an update. Physiol Rev 93, 359–404, doi: 10.1152/physrev.00033.2011 (2013). 23303913

[b15] Cohen A. . Abdominal fat is associated with lower bone formation and inferior bone quality in healthy premenopausal women: a transiliac bone biopsy study. J Clin Endocrinol Metab 98, 2562–2572, doi: 10.1210/jc.2013-1047 (2013). 23515452 PMC3667251

[b16] Junior I. F. . The relationship between visceral fat thickness and bone mineral density in sedentary obese children and adolescents. BMC Pediatr 13, 37, doi: 10.1186/1471-2431-13-37 (2013). 23510224 PMC3606829

[b17] Yu L. . Adiponectin regulates bone marrow mesenchymal stem cell niche through a unique signal transduction pathway: an approach for treating bone disease in diabetes. Stem Cells 33, 240–252, doi: 10.1002/stem.1844 (2015). 25187480 PMC4681406

[b18] Xie H. . Insulin-like effects of visfatin on human osteoblasts. Calcif Tissue Int 80, 201–210, doi: 10.1007/s00223-006-0155-7 (2007). 17340225

[b19] Wu S. S. . Omentin-1 Stimulates Human Osteoblast Proliferation through PI3K/Akt Signal Pathway. Int J Endocrinol 2013, 368970, doi: 10.1155/2013/368970 (2013). 23606838 PMC3626246

[b20] Xie H. . Omentin-1 exerts bone-sparing effect in ovariectomized mice. Osteoporos Int 23, 1425–1436, doi: 10.1007/s00198-011-1697-8 (2012). 21755404

[b21] Thommesen L. . Expression and regulation of resistin in osteoblasts and osteoclasts indicate a role in bone metabolism. J Cell Biochem 99, 824–834, doi: 10.1002/jcb.20915 (2006). 16721825

[b22] Nakatsuka A. . Visceral adipose tissue-derived serine proteinase inhibitor inhibits apoptosis of endothelial cells as a ligand for the cell-surface GRP78/voltage-dependent anion channel complex. Circ Res 112, 771–780, doi: 10.1161/CIRCRESAHA.111.300049 (2013). 23307819

[b23] Nakatsuka A. . Vaspin is an adipokine ameliorating ER stress in obesity as a ligand for cell-surface GRP78/MTJ-1 complex. Diabetes 61, 2823–2832, doi: 10.2337/db12-0232 (2012). 22837305 PMC3478540

[b24] Esteghamati A. . Gender-dependent effects of metformin on vaspin and adiponectin in type 2 diabetes patients: a randomized clinical trial. Horm Metab Res 45, 319–325, doi: 10.1055/s-0032-1330008 (2013). 23225237

[b25] Korner A. . Vaspin is related to gender, puberty and deteriorating insulin sensitivity in children. Int J Obes (Lond) 35, 578–586, doi: 10.1038/ijo.2010.196 (2011). 20856257

[b26] Shaker O. G. & Sadik N. A. Vaspin gene in rat adipose tissue: relation to obesity-induced insulin resistance. Mol Cell Biochem 373, 229–239, doi: 10.1007/s11010-012-1494-5 (2013). 23135683

[b27] Fazeli M. S. . Circulating levels of novel adipocytokines in patients with colorectal cancer. Cytokine 62, 81–85, doi: 10.1016/j.cyto.2013.02.012 (2013). 23474107

[b28] Li H. L. . Association of vaspin gene polymorphisms with coronary artery disease in Chinese population and function study. Clin Chim Acta 415, 233–238, doi: 10.1016/j.cca.2012.10.042 (2013). 23123830

[b29] Kukla M. . Serum vaspin may be a good indicator of fibrosis in chronic hepatitis C and is not altered by antiviral therapy. Pol J Pathol 63, 213–220 (2012). 23359189 10.5114/pjp.2012.32767

[b30] Kamio N. . Vaspin attenuates RANKL-induced osteoclast formation in RAW264.7 cells. Connect Tissue Res 54, 147–152, doi: 10.3109/03008207.2012.761978 (2013). 23323745

[b31] Zhu X. . Vaspin attenuates the apoptosis of human osteoblasts through ERK signaling pathway. Amino Acids 44, 961–968, doi: 10.1007/s00726-012-1425-5 (2013). 23135225

[b32] Guan X. . miR-223 Regulates Adipogenic and Osteogenic Differentiation of Mesenchymal Stem Cells Through a C/EBPs/miR-223/FGFR2 Regulatory Feedback Loop. Stem Cells 33, 1589–1600, doi: 10.1002/stem.1947 (2015). 25641499

[b33] Satue M., Arriero Mdel M., Monjo M. & Ramis J. M. Quercitrin and taxifolin stimulate osteoblast differentiation in MC3T3-E1 cells and inhibit osteoclastogenesis in RAW 264.7 cells. Biochem Pharmacol 86, 1476–1486, doi: 10.1016/j.bcp.2013.09.009 (2013). 24060614

[b34] Zhang Y. . A program of microRNAs controls osteogenic lineage progression by targeting transcription factor Runx2. Proc Natl Acad Sci U S A 108, 9863–9868, doi: 10.1073/pnas.1018493108 (2011). 21628588 PMC3116419

[b35] Zhang Y. . Hippocalcin-like 1 suppresses hepatocellular carcinoma progression by promoting p21 stabilization via activating ERK1/2-MAPK pathway. Hepatology, doi: 10.1002/hep.28395 (2015). 26659654

[b36] Yu J. S. . PI3K/mTORC2 regulates TGF-beta/Activin signalling by modulating Smad2/3 activity via linker phosphorylation. Nat Commun 6, 7212, doi: 10.1038/ncomms8212 (2015). 25998442 PMC4455068

[b37] Fujita T. . Runx2 induces osteoblast and chondrocyte differentiation and enhances their migration by coupling with PI3K-Akt signaling. J Cell Biol 166, 85–95, doi: 10.1083/jcb.200401138 (2004). 15226309 PMC2172136

[b38] Feng Y. . Exendin-4 promotes proliferation and differentiation of MC3T3-E1 osteoblasts by MAPK activation. J Mol Endocrinol, doi: 10.1530/JME-15-0264 (2015). 26647389

[b39] Zhang W. . Lactoferrin stimulates osteoblast differentiation through PKA and p38 pathways independent of lactoferrin’s receptor LRP1. J Bone Miner Res 29, 1232–1243 (2014). 24877241 10.1002/jbmr.2116

[b40] Blahna M. T. & Hata A. Regulation of miRNA biogenesis as an integrated component of growth factor signaling. Curr Opin Cell Biol 25, 233–240, doi: 10.1016/j.ceb.2012.12.005 (2013). 23312066 PMC4429755

[b41] Shibasaki S. . Blocking c-Met signaling enhances bone morphogenetic protein-2-induced osteoblast differentiation. FEBS Open Bio 5, 341–347, doi: 10.1016/j.fob.2015.04.008 (2015). PMC4415006 25941631

[b42] Goyal L., Muzumdar M. D. & Zhu A. X. Targeting the HGF/c-MET pathway in hepatocellular carcinoma. Clin Cancer Res 19, 2310–2318, doi: 10.1158/1078-0432.CCR-12-2791 (2013). 23388504 PMC4583193

[b43] Li Y. Q. . MiR-34c suppresses tumor growth and metastasis in nasopharyngeal carcinoma by targeting MET. Cell Death Dis 6, e1618, doi: 10.1038/cddis.2014.582 (2015). 25611392 PMC4669777

[b44] Tanaka N. . Downregulation of microRNA-34 induces cell proliferation and invasion of human mesothelial cells. Oncol Rep 29, 2169–2174, doi: 10.3892/or.2013.2351 (2013). 23525472

[b45] Cai K. M. . Hsa-miR-34c suppresses growth and invasion of human laryngeal carcinoma cells via targeting c-Met. Int J Mol Med 25, 565–571 (2010). 20198305 10.3892/ijmm_00000378

[b46] Liu Y. . Novel adipokines and bone metabolism. Int J Endocrinol 2013, 895045, doi: 10.1155/2013/895045 (2013). 23431296 PMC3575660

[b47] Huang C. Y. . Adiponectin increases BMP-2 expression in osteoblasts via AdipoR receptor signaling pathway. J Cell Physiol 224, 475–483, doi: 10.1002/jcp.22145 (2010). 20432444

[b48] Biver E. . Influence of adipokines and ghrelin on bone mineral density and fracture risk: a systematic review and meta-analysis. J Clin Endocrinol Metab 96, 2703–2713, doi: 10.1210/jc.2011-0047 (2011). 21778223

[b49] Bertoni L. . Leptin increases growth of primary ossification centers in fetal mice. J Anat 215, 577–583, doi: 10.1111/j.1469-7580.2009.01134.x (2009). 19682137 PMC2780574

[b50] Yadav V. K. . A serotonin-dependent mechanism explains the leptin regulation of bone mass, appetite, and energy expenditure. Cell 138, 976–989, doi: 10.1016/j.cell.2009.06.051 (2009). 19737523 PMC2768582

[b51] Kang H. J. . Involvement of miR-34c in high glucose-insulted mesenchymal stem cells leads to inefficient therapeutic effect on myocardial infarction. Cell Signal 27, 2241–2251, doi: 10.1016/j.cellsig.2015.07.024 (2015). 26232617

[b52] Bhatnagar S. . Increased microRNA-34c abundance in Alzheimer’s disease circulating blood plasma. Front Mol Neurosci 7, 2, doi: 10.3389/fnmol.2014.00002 (2014). 24550773 PMC3912349

[b53] Morizane R. . miR-34c attenuates epithelial-mesenchymal transition and kidney fibrosis with ureteral obstruction. Sci Rep 4, 4578, doi: 10.1038/srep04578 (2014). 24694752 PMC3974136

[b54] Palmieri A. . Comparison between titanium and anatase miRNAs regulation. Nanomedicine 3, 138–143, doi: 10.1016/j.nano.2007.03.004 (2007). 17572356

[b55] Bae Y. . miRNA-34c regulates Notch signaling during bone development. Hum Mol Genet 21, 2991–3000, doi: 10.1093/hmg/dds129 (2012). 22498974 PMC3373245

[b56] Ducy P., Zhang R., Geoffroy V., Ridall A. L. & Karsenty G. Osf2/Cbfa1: a transcriptional activator of osteoblast differentiation. Cell 89, 747–754 (1997). 9182762 10.1016/s0092-8674(00)80257-3

[b57] Komori T. . Targeted disruption of Cbfa1 results in a complete lack of bone formation owing to maturational arrest of osteoblasts. Cell 89, 755–764 (1997). 9182763 10.1016/s0092-8674(00)80258-5

[b58] van der Deen M. . MicroRNA-34c inversely couples the biological functions of the runt-related transcription factor RUNX2 and the tumor suppressor p53 in osteosarcoma. J Biol Chem 288, 21307–21319, doi: 10.1074/jbc.M112.445890 (2013). 23720736 PMC3774399

[b59] Lin S. & Gregory R. I. MicroRNA biogenesis pathways in cancer. Nat Rev Cancer 15, 321–333, doi: 10.1038/nrc3932 (2015). 25998712 PMC4859809

[b60] Trabucchi M. . The RNA-binding protein KSRP promotes the biogenesis of a subset of microRNAs. Nature 459, 1010–1014, doi: 10.1038/nature08025 (2009). 19458619 PMC2768332

[b61] Trusolino L., Bertotti A. & Comoglio P. M. MET signalling: principles and functions in development, organ regeneration and cancer. Nat Rev Mol Cell Biol 11, 834–848, doi: 10.1038/nrm3012 (2010). 21102609

[b62] Huang J. . miR-199a-3p inhibits hepatocyte growth factor/c-Met signaling in renal cancer carcinoma. Tumour Biol 35, 5833–5843, doi: 10.1007/s13277-014-1774-7 (2014). 24609899

[b63] Liu G. Y. . Leptin promotes the osteoblastic differentiation of vascular smooth muscle cells from female mice by increasing RANKL expression. Endocrinology 155, 558–567, doi: 10.1210/en.2013-1298 (2014). 24248461

[b64] Cui R. R. . MicroRNA-204 regulates vascular smooth muscle cell calcification *in vitro* and *in vivo*. Cardiovasc Res 96, 320–329, doi: 10.1093/cvr/cvs258 (2012). 22871591

[b65] Cheung O. . Nonalcoholic steatohepatitis is associated with altered hepatic MicroRNA expression. Hepatology 48, 1810–1820, doi: 10.1002/hep.22569 (2008). 19030170 PMC2717729

